# Waterloo Better Beginnings as a Transformative Prevention Project: Impacts on Children, Parents, and the Community

**DOI:** 10.3390/ijerph17103442

**Published:** 2020-05-15

**Authors:** Geoffrey Nelson, Julian Hasford, Carlos Luis Zatarain, Alexis Gilmer, S. Kathleen Worton, Marwa Eid, Salma Bangash, Jeremy Horne

**Affiliations:** 1 Department of Psychology, Wilfrid Laurier University, Waterloo, ON N2L 3C5, Canada; carlosluisz@gmail.com (C.L.Z.); gilm3440@mylaurier.ca (A.G.); skworton@gmail.com (S.K.W.); 2 School of Child and Youth Care, Ryerson University, Toronto, ON M5B 2K3, Canada; jhasford@ryerson.ca; 3 Better Beginnings Waterloo, Adventure4Change, Waterloo, ON N2L 6C7, Canada; marwasobhi74@yahoo.com (M.E.); s_bangash@live.com (S.B.); Jeremy@adventure4change.org (J.H.)

**Keywords:** prevention, early childhood development, children’s mental health, evaluation

## Abstract

Better Beginnings Waterloo (BBW) is an ecological, community-driven, prevention program for children aged 4–8 and their families. BBW was implemented in two low-income communities with high percentages of visible minorities. Data on Grade 1–2 children and their parents (the baseline comparison group) were gathered through parent interviews (*n* = 34) and teacher reports (*n* = 68) in 2015, prior to BBW programs, and in the period 2018–2019, the same data were collected through parent interviews (*n* = 47) and teacher reports (*n* = 46) for children and parents participating in programs (the BBW group). As well, qualitative, open-ended individual interviews with parents (*n* = 47) and two focus groups were conducted in the period 2018–2019. Children in the BBW cohort were rated by their teachers as having a significantly lower level of emotional and behavioural problems than those in the baseline sample; parents in the BBW cohort had significantly higher levels of social support than parents in the baseline cohort; BBW parents rated their communities significantly more positively than parents at baseline. The qualitative data confirmed these findings. The quantitative and qualitative short-term findings from the BBW research showed similar positive impacts to previous research on program effectiveness, thus demonstrating that the Better Beginnings model can be successfully transferred to new communities.

## 1. Introduction

Better Beginnings, Better Futures (BBBF) is a model for the prevention of mental health problems among children in marginalized communities. We begin by noting the problem that BBBF addresses, the BBBF model, and the original research in three communities on the effectiveness of BBBF. 

### 1.1. Children’s Mental Health as a Public Health Problem

It has long been recognized that children’s mental health is a significant public health problem [1]. In Ontario, a large epidemiological study of 5785 children aged 4–16 conducted in 2014 found that 19.6% had a mental disorder [2]. Children’s mental health problems often persist into adulthood, and there will never be enough mental health resources to solve this problem through treatment. Rather, as Waddell et al. [1] have argued, only prevention can reduce this high rate of mental disorders among children.

Ethnic minorities comprise approximately 30% of Ontario’s population (with rates exceeding 50% in some urban centres of Southern Ontario), with South Asian, Chinese, and Black populations comprising the largest groups [3]. Existing evidence suggests that immigrant children may have a lower prevalence of mental disorder than their Canadian-born counterparts [4], but that prevalence rates vary considerably based upon the ethnicity or country of origin [5], migration status [6], regional context [7], and prejudice and linguistic fluency [8]. Moreover, there is considerable evidence that ethnic minorities in Canada face considerable inequities across multiple social determinants of mental health [9], in areas such as employment [10], housing [11], and mental service utilization [12].

### 1.2. The Better Beginnings, Better Futures Model

In Ontario, BBBF was developed in the 1990s as a prevention model to address children’s mental health [13,14]. The most distinguishing characteristic of BBBF, compared to other prevention models, is its focus on resident participation. In BBBF, the role of residents is transformed from program participant to the multiple roles of board member, committee member, program volunteer, and staff, having input on what programs are offered and how the programs are managed and delivered. The focus on resident participation is consistent with an empowerment-oriented, asset-based, community-building approach to prevention [15]. 

The original BBBF was implemented in three low-income Ontario communities for children aged 4–8 and their families. It has been argued that low-income communities should be the focus of community-wide prevention programs that aim to promote multiple domains of children’s health and well-being by targeting multiple ecological levels [16]. Moreover, the three communities are diverse, with predominantly Francophone residents in one community (Cornwall), a substantial representation of Indigenous people in another community (Sudbury), and a multi-culturally diverse community of new Canadians in the North Etobicoke area of Metropolitan Toronto. 

The BBBF model is guided by a set of principles [13]. It is: 

(1) *Ecological and holistic*, with neither one single focus nor one single program, but a broad focus on many different programs that address the whole child, the family, the school, and the community; 

(2) *Community driven*, with local residents playing a central role in project governance, program planning, and program implementation; 

(3) *Integrated and partnership based*, with programs that “blend and unite” existing educational, health, social, and community services; and

(4) *Prevention focused and universally available* to all children aged 4–8 and their families in the community. 

While BBBF programs must adhere to these principles, local stakeholders have considerable latitude in deciding what programs to offer. Thus, programs can be tailored to the local context. In line with the ecologically-oriented, community-driven approach to primary prevention, the three main goals of BBBF are:

(1) To prevent children’s emotional and behavioural problems and to promote positive child development; 

(2) To enhance resident participation and positive outcomes for parents and families; 

(3) To promote community development and improve community well-being. 

### 1.3. Better Beginnings Research

Research was undertaken to evaluate the impacts of the original three BBBF projects on children, parents/families, and communities [13]. Two designs were used: a baseline-focal cohort design and a longitudinal comparison group design. In the baseline-focal cohort design, children in Grade 2 prior to the implementation of BBBF programs were compared with children in Grade 2 after 1–4 years of participation in BBBF programs [17]. The main findings from this research on short-term outcomes were that the BBBF children showed lower rates of overanxious behaviour and higher rates of self-control, according to teachers; the BBBF parents showed lower levels of smoking and stressful life events, and higher marital satisfaction; and BBBF parents reported improved relationships and involvement with their child’s school.

In the longitudinal comparison group design, children and their families who participated in BBBF were assessed annually (Junior Kindergarten [age 4], Senior Kindergarten [age 5], Grades 1 [age 6] and 2 [age 7]), as they progressed through the four years of the project, and were compared to children and families from two matched comparison communities. Adding a following cohort after the Grade 2 data collection to increase the sample size, participants from the BBBF and comparison communities were again assessed when children were in Grades 3, 6, and 9 to determine the medium-term impacts of the project. Positive impacts of BBBF were found for children’s school and social functioning and emotional and behavioural problems; and BBBF parents reported greater social support, marital satisfaction, family functioning, and more positive neighbourhood impacts [13]. 

The longitudinal cohort was followed up until age 18 to determine the long-term impacts of BBBF. A lower percentage of children from BBBF communities had been involved in special education, and BBBF children showed higher grades in high school, reported more regular exercise, and fewer reported committing property offences [18]. Parents from the BBBF communities reported less alcohol consumption, less smoking in the home, had lower levels of clinical depression, and were more involved in their communities than parents from the comparison communities [19]. BBBF youth rated their communities as safer and more free from crime than youth from comparison communities [19]. Moreover, it is important to note that these impacts were found in communities with varying types of diversity, including one multi-racial and multi-ethnic community with a preponderance of immigrants from the Caribbean and southern Asia and another community with a substantial Indigenous population. 

Data on 19 government service cost measures were collected from the longitudinal research sample from the time the children were in JK to Grade 12 (18 years old), 10 years after ending project participation [18]. When the BBBF monetary return on investment as a ratio of savings to costs was calculated, a return of $2.50 per family for every $1 invested was found by Grade 12. 

In the remainder of the paper, we describe the impacts of a recently created program—Better Beginnings Waterloo (BBW)—on children, families, and the community. BBW was modeled after the original BBBF programs. We aimed to determine whether the BBBF model could be successfully transferred to new communities without the same level of funding and administrative oversight as was provided to the three original BBBF demonstration project communities.

### 1.4. Hypotheses

The hypotheses of this research were as follows:

(1) The BBW group will show better outcomes than the baseline comparison group on the quantitative measures and qualitative indicators of children’s mental health; 

(2) The BBW group will show better outcomes than the baseline comparison group on the quantitative measures and qualitative indicators of parent/family well-being, and 

(3) The BBW group will show better outcomes than the baseline comparison group on the quantitative measures and qualitative indicators of community well-being.

## 2. Materials and Methods 

### 2.1. Context and BBW Program

As part of a pan-Canadian knowledge transfer initiative [20], Better Beginnings Waterloo (BBW) was created and implemented in two adjacent school communities (Cedarbrae and Winston Churchill) in Waterloo, Ontario. These communities are characterized by low levels of income (15–17% lived below Canada’s Low-Income Cut Off) and high percentages (35–42%) of visible minorities [3]. 

BBW programs for children in Grades Junior Kindergarten (age 4) to Grade 2 (age 7) were created based on the input of parents and school personnel. Through the introduction of Action Teams that consist of volunteers and staff, residents have ongoing opportunities to suggest new programs and shape and direct existing ones. Furthermore, BBW staff practice assertive outreach [21] to engage residents in program activities and to encourage them to adopt volunteer roles. 

Consistent with the ecological thrust of the BBBF model, there were programs for children, families, and the community. Child-focused programs include Running and Reading at Cedarbrae, Run and Learn at Winston Churchill, a home reading program for Cedarbrae students, music, dance, sports, nutrition, and yoga programs, a tutoring program, and March break and summer camps and programs. For parents and families, there are parents’ groups at Cedarbrae and in the community, family days, family trips, English classes, school readiness, and parents’ night out. Community activities consist of a variety of events, including a program with the local symphony, and community and school-based celebrations. Programs are offered at the Hub (a store-front centre located mid-way between the two schools) and in the schools. However, most school-based programs are offered to Cedarbrae students and parents, as Winston Churchill has less accessible space for programs.

### 2.2. Evaluation Design and Methods

A mixed-methods approach [22], including quantitative and qualitative methods, was used. While quantitative research uses numerical data and statistical analyses, qualitative research uses non-numerical data and focuses on meaning making [23]. The particular type of mixed-methods design used is the triangulation design, in which the researcher seeks to triangulate the findings of quantitative and qualitative data [23]. 

### 2.3. Quantitative Methods

**Design.** A baseline comparison group–BBW group design was used, as in the original BBBF research [17]. Grade 1 and 2 teachers sent home letters and consent forms, and parents were also recruited from BBW programs and events. Baseline data on the children and parents were gathered through parent interviews (*n* = 34) and teacher reports (*n* = 68) in 2015, and in the period 2018–2019, the same data were collected through parent interviews (*n* = 47) and teacher reports (*n* = 46) for children and parents participating in BBW. 

**Participants.** On average, the parents had lived in their neighbourhoods for 4–5 years. The parents were in their late 30s or early 40s, while the children were 8–10 years of age. The vast majority of the interviews (> 90%) were completed with the mothers of the children. The parents had completed on average 9–11 years of education, and the monthly family income averaged approximately $3200 Canadian. The samples had relatively equal numbers of boys and girls. The majority of the parents had been or were currently married, and the majority of participants were born outside of Canada, with most immigrating from Somalia, Middle-Eastern countries, India, Pakistan, and China. The baseline and BBW samples did not differ significantly on years lived in the community, parents’ educational levels, monthly income, sex of the child, parents’ marital status, and languages spoken at home. The two groups did differ significantly on age of the parent, *t* (79) = 2.27, *p* = 0.03, and age of the child, *t* (78) = 14.57, *p* = 0.001, with parents and children in the baseline group being older than parents and children in the BBW group (see Table 1). 

Cedarbrae participants scored higher than Winston Churchill participants on a measure of the number of times that parents and families participated in BBW programs and activities, *t* (42) = 2.24, *p* = 0.03. 

**Instruments.** Parent-rated measures were taken from the original BBBF research [17]. Children’s social skills were measured with Gresham and Elliot’s [24] 39-item Social Skills Rating Scale. Each item (e.g., “helps with household tasks without being asked,” “appropriately questions household rules that may be unfair”) is rated on a three-point scale from “never” to “very often.” Cronbach’s alpha for this measure was α = 0.93. Emotional and behavioural problems were measured with a 30-item scale from the Revised Ontario Child Health Study [25]. Each item (e.g., “temper tantrums or hot temper,” “impulsive, acts without thinking”) is rated on a three-point scale from “never or not true” to “often or very true.” Cronbach’s alpha was α = 0.92. 

The Social Provisions Scale was used to measure parents’ social support [26]. The five items (e.g., “there are people I can count on in an emergency”) are rated on a four-point scale from “strongly agree” to “strongly disagree.” Cronbach’s alpha was α = 0.70. There was a 15-item list of stressful life events [17]. The 24-item Center for Epidemiologic Studies Depression scale was used to measure parental depression [27]. Items (e.g., “I felt sad”) are rated on a four-point scale from “rarely or none of the time” to “most or all of the time.” Cronbach’s alpha was α = 0.91. A nine-item measure of neighbourhood satisfaction was used. Items (e.g., “safety walking on the street at night”) are rated on a five-point scale from “excellent” to “poor,” with a low score indicating a high level of satisfaction. Cronbach’s alpha was α = 0.78.

The teacher report form included a 30-item version of the Social Skills Rating Scale, similar in format to the parent measure of social skills. Cronbach’s alpha was α = 0.95. There was also a 64-item measure of children’s emotional and behavioural problems, similar in format to the parent-rated measure. Cronbach’s alpha was α = 0.95. Additionally, there was a four-item measure (reading, spelling, writing, mathematics) of academic achievement rated on a five-point scale from “far below grade” to “far above grade.” Cronbach’s alpha was α = 0.92. 

**Procedure.** Baseline comparison parent interviews were conducted by a female community researcher hired and trained by the research team. A different female community researcher completed all the parent interviews for the BBW group. Baseline and BBW group parent interviews lasted from 40 min to 150 min, averaging 69 minutes per interview. Parents were reimbursed $25 for the baseline comparison group interviews and $30 for BBW group interviews. Parents provided their informed consent to do the interview. 

Teachers were asked to complete a report for all Grade 1 and 2 children whose parents provided consent. Teachers were reimbursed $20 per rating form. One member of the research team communicated with the teachers about when and how to complete the teacher reports. All of the teacher reports were completed online. 

**Data analysis.** In view of the previously mentioned difference in levels of participation for parents from the two schools, the data for children and parents from Cedarbrae and Winston Churchill were analyzed separately using independent sample *t*-tests to compare the baseline comparison group and BBW samples on the outcome measures. The Statistical Package for the Social Sciences (SPSS) was used to compute the statistical tests. 

### 2.4. Qualitative Methods

**Design.** As part of the mixed-methods triangulation design, the participants in the BBW sample of parents were asked to participate in open-ended qualitative interviews. The interviews explored outcomes of their participation in BBW. The questions were designed to tap the same outcomes as the quantitative measures, namely child, parent/family, and community outcomes. 

**Participants.** In the period 2018–2019, the researchers conducted qualitative interviews with parents (*n* = 47) of Grade 1 and 2 children who also participated in the quantitative interviews. As well, parents who volunteered in BBW (*n* = 3) and parents who participated in a parents’ group (*n* = 4) were interviewed in two separate focus group interviews. 

**Instruments.** The individual parent interviews included qualitative, open-ended questions about program impacts. Sample questions are as follows:

“What impact did participating in Better Beginnings have on you as an individual?”

“What impact did participating in Better Beginnings have on your child{ren)?”

“What impact did participating in Better Beginnings have on your community?”

The two focus group interviews paralleled the individual parent interviews with questions concerning the impacts of participating in BBW. 

**Procedure.** The individual parent interviews were conducted by the same community researcher who conducted the quantitative parent interviews. The focus groups were conducted by graduate students in Community Psychology. 

**Data analysis.** Two researchers independently coded the focus group transcripts and the responses from the qualitative open-ended questions from the individual parent interviews. The researchers used the NVivo 12 qualitative analysis software package [28] for the analysis. Having identified common themes from the data, the researchers met with the community researcher who served as a check on the trustworthiness of the findings.

## 3. Results

### 3.1. Children

**Quantitative findings.** Children in the BBW sample at the school where were concentrated (Cedarbrae) were rated by their teachers as having a significantly lower level of emotional and behavioural problems than those in the baseline sample, *t* (45) = 2.78, *p* = 0.008, but there were no significant effects on social skills or academic achievement (see Table 2). There were also no significant findings on parent-rated social skills and emotional and behavioural problems. 

**Qualitative findings.***Reduced emotional and behavioural problems* was also a theme in the qualitative research. As one parent stated, “(my child) suffers from anxiety. Participating with BBW helped her a lot emotionally. I can see a big difference,” while another parent reported that her daughter became “better in controlling her temper.”

Parents also noted *enhanced social skills and improved self-confidence through positive social interactions* amongst their children. For example, one parent said that BBW activities “helped my kids to socialize, getting out of their comfort zone.” A number of parents described how children overcame shyness through “the programs [that] give kids more chances for social interactions and recognize each other.” As one parent shared:

“Yeah, before the school would say ‘why [is] she scared?’ Even when I bring the children to school, like she’s scared still outside. But now she’s good. She’ll play outside with kids, she brings her sister and brother outside.”

Some parents also noted *improved academic skills* of their children, especially in reading.

“There was one kid that all year was waiting to get to level J in the reading. She started off I think in level C or D, somewhere around there. And there was a book that she wanted to read in that J bin, more than anything. And every time she’d walk down there she’d look at it and point and say ‘I’m gonna’ get to read that.’ And she did. And the day that she got to read it, it was just the most exciting thing.”

(Parent, focus group)

“They think they joined Better Beginnings programs to have fun, but in fact they joined it to learn. It’s just we switch it for them… But Better Beginnings, yes they want to go because they think they’re gonna’ have fun and they do have fun, but they learn at the same time. We just put it in the right context for them, that’s why they love joining the programs.”

(Parent, focus group)

### 3.2. Parents/Families

**Quantitative findings.** There was no significant difference on the measure of depression between the baseline and BBW sample (see Table 3). However, for Cedarbrae parents, the BBW group scored significantly higher than the baseline group on the Social Provisions Scale, *t* (34) = 2.97, *p* = 0.05. Further, the Winston Churchill BBW sample showed significantly lower levels of stressful life events compared with the baseline Winston Churchill sample; *t* (14) = 3.48, *p* = 0.004.

**Qualitative findings.** Themes in the qualitative data regarding impacts on parents suggested that participating in Better Beginnings helped parents to reduce isolation and *increase social connectedness through new friendships with other parents*. As one parent shared:

“[Better Beginnings has had] a big impact. If it were not for BBW I would not have that much support from which I consider a family.”

(Parent, individual interview)

For several parents, this increased social connectedness helped to improve their mental health, particularly through *reduced depression/anxiety*:

“… For me … I learned from the program…instead of sitting in the house and being depressed all the time, I leave the house, I make friends, I meet a lot of people, I laugh with them, I talk with them… They give me advice and I give them advice… when we sit at home we have no one to ask. We don’t have family here…”

(Parent, focus group)

“They brought me out of my bubble. I was stuck home all the time suffering from anxiety. Now I have a different attitude and I have a purpose.”

(Parent, individual interview)

BBW staff witnessed a growth in *resident empowerment*, including knowledge, skills, improvement in English language skills, and personal growth. Early on, a mother of seven children said to one of the staff: “I am poor, I was a afraid to speak up.” That is changing for some women who have gone back to school for retraining and who are carving out careers for themselves. The previously mentioned Action Teams have been catalysts for resident empowerment. Many participants described the personal growth they experienced through BBW. One shared that: 

“It changed my life. I wake up every day with something to do every day. I gained a lot of friends. It taught me a lot of techniques to use with my kids.”

Another parent reported that:

“Involvement with kids gave me a lot more options to work on my field …, like other ways. And being involved, I’m not employed right now but like I can see hundreds of projects, hundreds of ideas coming out of my brain. It’s just like jumping around and say ‘yes, yes, yes, yes.’”

### 3.3. Community

**Quantitative findings.** For Cedarbrae parents, but not parents from Winston Churchill, the measure of neighbourhood satisfaction was significantly lower for the BBW group than for the baseline group, *t* (34) = 2.12, *p* = 0.041, indicating that BBW parents were more satisfied with their community than parents at baseline (see Table 3).

**Qualitative findings.** Parents also spoke of the impacts of BBW on the community in the qualitative interviews. These impacts included *community building, cultural bridging*, and *safety*. Community building referred to the ways in which the events and physical spaces offered by BBW facilitated connections that “bring everybody together.” Participants recognized the benefits of such community building:

“[The impact BBW has had in the community is] to bring people together eating, playing, and having fun.”

(Parent, individual interview)

“It is an opportunity to give everyone a chance to be involved. Opening doors for everyone, even if you think you cannot.”

(Parent, individual interview)

“We have lived in Sunnydale four years and half. We didn’t know each other even when we started the program, Better Beginnings. Now I know all my neighbours, my kids’ friends, moms, I know them. Before I say I knew only one kid. … Now when we come in the morning we know each other. Yeah it’s good.”

(Parent, individual interview)

Cultural bridging was a second major community impact, which involved the facilitation of interactions between residents from different cultural and ethnic backgrounds. BBW activities were reported to create “better connection between different races. [Where] kids playing together no matter what your background is,” and “a chance to see people from different cultures having fun together”.

“(BBW) brought people together. The Hub is not just a place to hang out, it is a place where people from different cultures and backgrounds can connect, forming a community.”

(Parent, individual interview)

Safety was also an important theme:

“Now we know that our kids are in a safe environment when they join BBW programs.”

(Parent, individual interview)

## 4. Discussion

An important issue in prevention science is whether effective program models can be scaled up to new communities and achieve the same positive impacts [29]. Demonstration projects often have ideal conditions for success—they are well funded, have high-quality and well-trained staff, and are subject to rigorous research that can aid implementation. While other Canadian communities indicated their intentions to adopt aspects of BBBF following a pan-Canadian knowledge transfer [20], BBW was the first new BBBF program that adopted the full model and had a major evaluation. Here, we discuss the main outcomes, the factors facilitating the success of the program, and the limitations of this research. 

### 4.1. Replication of Program Outcomes

**Child outcomes.** While there was a positive effect for decreased teacher-rated overanxious behaviour in the baseline-focal analysis of the original BBBF research [17], BBW children at Cedarbrae showed a significant reduction on an overall measure of emotional and behavioural problems. The qualitative data from parents also revealed a theme of reduced emotional and behavioural problems for children, which provides confirmation of the quantitative finding. Moreover, the fact that this effect was achieved only at Cedarbrae, where programs were concentrated, shows that there must be a sufficient program “dosage” to achieve such an effect [30]. These findings are also consistent with a large body of prevention research that has demonstrated that prevention programs for young children are particularly effective for minority families [30]. 

Further, in the original research, there was a positive effect on children’s social skills at one of the sites. This site had implemented a school-wide social skills program as part of its BBBF programs. The other original sites and the two schools in the BBW project did not have such a program, which may be necessary for achieving a positive impact on children’s social skills. In the BBW research, while there was no significant impact on the quantitative measure of social skills, the parents mentioned improvements in their children’s social skills in the qualitative interviews. Perhaps the lack of convergence between the quantitative and qualitative data in this regard reflects the fact that this impact was limited to some parents, but not all parents in the community. 

Finally, like the original research, BBW did not show a significant impact on children’s academic achievement. While improved academic achievement was a theme in the qualitative findings, this again may be restricted to a small number of parents. In the original research, academic impacts did not become evident until Grade 6 and became stronger over time (Grades 9 and 12). Perhaps more time is required to observe academic impacts. It is also possible that the brief teacher rating measure of students’ achievement was not sensitive enough to detect effects. The lack of observed academic impacts may also be attributed to a high immigrant population within the communities, since some evidence suggests that newcomer elementary school students may demonstrate lower academic achievement due to factors such as language and acculturative stress [31]. 

**Parent/family outcomes.** There was a strong convergence between the quantitative and qualitative findings regarding enhanced social support. This finding is consistent with that from the original BBBF quantitative [17] and qualitative [32] research. The BBBF model emphasizes parent support groups and providing parents with multiple opportunities for socializing with other parents. 

While there was no significant impact on the measure of parental depression, parents mentioned reduced anxiety and depression in the qualitative interviews, often in the context of enhanced social support. In the original BBBF research, impacts on parental depression were not reported until Grades 9 and 12. It is noteworthy that many participants—the majority of whom were immigrant mothers—emphasized the importance of reduced social isolation, which is consistent with previous research that highlights the importance of social support in the well-being of newcomer women [33]. 

There was a significant reduction in stressful life events for Winston Churchill parents. In the original BBBF research, the reduction in stressful life events was observed primarily at one site [17]. While Winston Churchill parents participated less in BBW than Cedarbrae parents, they did participate in BBW parent and family activities that may have helped to reduce life stressors. Not enough parents at Cedarbrae completed the stressful life events measure to be able to analyze differences between the two samples. 

**Community outcomes.** In the BBW research, there was convergence in the finding of enhanced satisfaction with the Cedarbrae community. Similar community effects were observed in the original BBBF quantitative [17] and qualitative [32] research. The qualitative data provided a more nuanced understanding of community satisfaction. First, residents of the BBW project saw BBW as a catalyst for community building [15]. This was important because the Cedarbrae neighbourhood had a stigmatized reputation for years as a “problem community.” 

Second, residents spoke of the cultural bridging that occurs. The community is quite diverse along ethnic, racial, and religious lines. These findings might help point toward important mechanisms by which BBW improves the mental health of newcomers in particular, given that acculturative integration [34] and neighbourhood social capital [35] are important protective factors for mental health. The BBW project, and its setting, the Neighbourhood Hub, were seen as providing opportunities and space for community members from different backgrounds to come together, get to know one another, and develop relationships and a sense of community. The Hub also provides a space for people to engage in activities within their own culture, including family celebrations and wakes. 

Finally, the project was viewed as providing safe spaces for children, parents, families, and community members to come together and for the promotion of a physically and psychologically safe environment.

### 4.2. Factors Facilitating Success

What accounts for the success of BBW in replicating the main short-term outcomes of BBBF? We believe that the principles of BBBF are important for scaling the model up to new communities. Some prevention programs follow a “cookie cutter” approach, in which communities must rigidly adhere to the original program model. In our pan-Canadian knowledge transfer [20], we found that communities liked the emphasis on resident participation and believed that was something they could incorporate into their own programs. Indeed, an empowerment-oriented, asset-based, community-building approach to prevention [15] seemed to be a good fit for the Cedarbrae and Winston Churchill school communities. 

Having a local grassroots organization as the sponsor for BBW, with a local leader who lives in the neighbourhood and who had credibility with community residents and the schools, was important for establishing trust and a community-oriented approach to prevention. As well, the BBBF principle of partnership was evident in the relationships between the project, the schools, a neighbourhood centre located within the heart of the Cedarbrae community, two nearby universities with multiple opportunities for students for thesis, practicum, and service-learning placements in BBBW, and several other partners. All of this took time and patience on the part of the project founders, who realized the importance of developing trust and earning the respect of community members and other partners. 

A key element of BBBW practice that helped to achieve successful outcomes was assertive outreach and parental engagement. It was particularly important to engage the mothers who are often isolated at home, living in poverty, not knowing their neighbours, unfamiliar with the Canadian education system, and lacking English language skills. Preventive interventions can be particularly effective for such families, but staff engagement strategies with such families are particularly important for building trust, relationships, and resident empowerment. Moreover, promoting resident participation in parenting and English language learning programs and project events in the community are other important ways to engage parents. Previous research has also pointed to the importance of parent engagement [21]. Specific obstacles that the project helped to overcome were low income, lack of transportation, and lack of child care. Project activities were offered at no charge; transportation was available as needed; and child care was provided when women attended programs. Project staff members also advocated and supported parents in their dealings with the schools, police, and social services.

Another resource included having a BBBF toolkit and video for staff training and education of community members and partners [36]. BBW also had one of the original BBBF researchers as a project consultant and evaluator. This researcher was also able to bring in post-doctoral fellows and graduate and undergraduate students to assist with research on BBW. 

The community-building approach of BBW also provides many opportunities for resident participation. For example, in 2019, there were 572 volunteers in programs and events: 75 parents, 186 other adults, and 311 youth. With this high level of participation comes a sense of “community ownership” over the project, as was reported in the original BBBF research [32]. Indeed, many of the youth in the program had participated in BBW activities when they were younger and returned as volunteers in order to give back to the community.

The high level of volunteerism and hiring people from the community as staff provided community members with opportunities for training and job experience. Many of the volunteers are immigrants or refugees from countries that are plagued by war, violence, gender oppression, religious persecution, and authoritarian regimes that create climates of fear. Coming to the “promised land” of Canada with projects like BBW is a transformative, liberatory experience for many newcomers.

Another aspect of the BBBF model that is important for the development of new projects is its flexibility. Rather than following a “cookie cutter” approach, the model can be adapted within certain parameters to the unique circumstances of communities. Barrera, Gonzalex Castro, and Steiker [37] have conceptualized different types of adaptation of prevention programs for ethnic communities, ranging from “home-grown” initiatives to cultural adaptations of evidence-based programs developed by researchers. BBBF is somewhat of a hybrid model that is not completely community driven, professionally driven, or researcher driven. The BBBF model is flexible enough to be adapted to multi-cultural communities with ethnic and racial diversity, but at the same time has a clear theory of change with a set of core principles [13,14]. Hawe, Shiell, and Riley [38] make an important distinction between the form and the function of a complex community intervention that is relevant here. Complex interventions like BBBF can vary in the form or the specific programs undertaken, while still maintaining fidelity to the core functions or principles of a model. All of the original BBBF programs had their own particular set of programs, but they all adhered to the principles of resident participation, an ecological approach, partnerships, and universal prevention. 

### 4.3. Limitations

This study is limited by its examination of short-term impacts. Furthermore, the baseline-focal cohort design has limitations in terms of its ability to control for factors that jeopardize internal validity (i.e., the extent to which factors other than BBW may account for positive outcomes). Finally, only one site with two school communities was examined and the sample sizes were small relative to the original three-site BBBF study.

## 5. Conclusions

In summary, BBW is the first new Better Beginnings program in Ontario since the original three BBBF programs were implemented in the 1990s. The quantitative and qualitative short-term findings from the BBW research showed similar positive impacts to those achieved in the original BBBF research. BBW is meeting the main goals of BBBF: prevention of children’s emotional and behavioural problems, the enhancement of parental well-being and participation, and the promotion of community development and community well-being. 

Prevention programs like BBW are even more important today than they were 30 years ago, as there is evidence that children’s mental health problems have become more prevalent, at least in Ontario [2]. BBBF is a good model for engaging low-income, culturally diverse communities in preventing children’s mental health problems, promoting parental engagement and resident empowerment, and enhancing low-income communities as nurturing environments for children [16].

**Ethics approval.** This research was reviewed and approved by the Research Ethics Board of Wilfrid Laurier University and the Waterloo Region District School Board.

## Figures and Tables

**Table 1:** Comparison of Baseline and Better Beginnings Waterloo (BBW) Focal Cohort Samples on Demographic Characteristics.

Variables	Baseline Comparison (*n* = 34)	BBW Group (*n* = 47)
Parent and Child Characteristics	Mean (SD) or *n* (%)	Mean (SD) or *n* (%)
Total months lived in BBW neighbourhood	57.58 (50.37)	50.79 (55.76)
Age of parent	40.15 (6.39)	37.74 (6.84)
Age of child	9.94 (.73)	8.22 (.89)
Years of elementary and high school successfully completed by the parent	9.83 (2.86)	11.39 (12.13)
Current monthly household income	3257.74 (2927.12)	3203.83 (1304.08)
Parent completing the interview (mother)	32 (94.1%)	44 (93.6%)
Sex of child (girl)	18 (52.9%)	24 (51.0%)
Parent ever married (yes)	30 (88.2%)	35 (74.4%)
Language other than English spoken at home (yes)	20 (58.8%)	29 (63.0%)
School (Cedarbrae)—parent interview	7 (21.8%)	29 (61.7%)
School (Cedarbrae)—teacher report	19 (27.9%)	33 (71.7%)

**Table 2:** Comparison of Baseline and BBW Focal Cohort Samples on Child Outcomes.

Child Outcomes	School	Baseline Group	BBW Focal Cohort
Measures	Cedarbrae or Winston Churchill	Mean (SD)	Mean (SD)
Emotional and behavioural problems total—parent-rated (potential range is 1–3; the higher the score, the more problems)	Cedarbrae	1.57 (0.41)	1.59 (0.38)
	Winston Churchill	1.68 (0.30)	1.64 (0.41)
Emotional and behavioural problems total—teacher-rated (potential range is 1–3; the higher the score, the more problems)	Cedarbrae	1.47 (0.24)	1.27 (0.23)
	Winston Churchill	1.34 (0.29)	1.33 (0.26)
Social skills total—parent-rated (potential range is 1–3; the higher the score, the greater the social skill)	Cedarbrae	1.45 (0.17)	1.39 (0.29)
	Winston Churchill	1.40 (0.21)	1.41 (0.27)
Social skills total—teacher-rated (potential range is 1–3; the higher the score, the greater the social skill)	Cedarbrae	2.21 (0.32)	2.33 (0.40)
	Winston Churchill	2.30 (0.44)	2.22 (0.26)
Academic achievement—teacher-rated (potential range is 1–5; the higher the score, the better the academic achievement)	Cedarbrae	2.57 (1.05)	2.27 (0.85)
	Winston Churchill	2.92 (1.01)	2.46 (0.79)

**Table 3:** Comparison of Baseline and BBW Focal Cohort Samples on Parent and Community Outcomes.

Parent and Community Outcomes	School	Baseline Group	BBW Focal Cohort
Measures	Cedarbrae or Winston Churchill	Mean (SD)	Mean (SD)
Social Provisions Scale (potential range is 1–4; the higher the score, the more social support)	Cedarbrae	1.40 (0.45)	1.80 (0.49)
	Winston Churchill	1.63 (0.45)	1.50 (0.41)
Center for Epidemiologic Studies Depression Scale (potential range is 1–4; the higher the score, the greater the level of depression)	Cedarbrae	1.51 (0.45)	1.7 (0.63)
	Winston Churchill	1.56 (0.47)	1.7 (0.43)
Stressful life events (potential range is 1–15; the higher the score, the more stressful life events)	Cedarbrae	NA	NA ^1^
	Winston Churchill	7.33 (4.18)	1.30 (2.79)
Neighbourhood satisfaction (potential range is 1–5; the lower the score, the more satisfaction with one’s neighbhourhood)	Cedarbrae Winston Churchill	3.22 (0.73) 2.87 (0.53)	2.80 (0.65) 3.30 (0.65)

^1^ There were insufficient baseline data for the Cedarbrae group to perform the analysis on this measure.
